# Phenotypic classification of variability of non-syndromic congenital cleft lip and jaw in Vorderwald × Montbéliarde cattle

**DOI:** 10.1186/s13028-015-0177-0

**Published:** 2015-12-15

**Authors:** Sina Reinartz, Maren Hellige, Karsten Feige, Peter Wenning, Ottmar Distl

**Affiliations:** University of Veterinary Medicine Hannover, Foundation, Institute for Animal Breeding and Genetics, Bünteweg 17p, 30559 Hannover, Germany; University of Veterinary Medicine Hannover, Foundation, Clinic for Horses, Bünteweg 9, 30559 Hannover, Germany; University of Veterinary Medicine Hannover, Foundation, Clinic for Cattle, Bischofsholer Damm 15, 30173 Hannover, Germany

**Keywords:** Vorderwald cattle, Cleft lip and jaw, Phenotypic variability, Orofacial malformation

## Abstract

**Background:**

Non-syndromic congenital cleft lip and jaw (CLJ) is a condition reported in several cattle breeds, but a detailed classification system does not exist for cattle. The objective of the present study was to describe the phenotypic variability of this orofacial malformation in Vorderwald × Montbéliarde cattle. For this purpose, a classification system of CLJ was developed on examination of five orofacial structures: (1) lips, (2) the *processus* (*proc.*) *nasalis* of the *os incisivum*, (3) the dental plate with adjacent segments of the hard palate, (4) the facial bones (*os incisivum*, *os maxillare*, *os nasale* and *os palatinum*) and (5) the mandibles. Each structure was given a score reflecting the degree of the lesion from absent (score 0) to severe.

**Results:**

Nine cases were included in the study and they shared absence of the abaxial rostral part of the *processus* (*proc.*) *nasalis* of the *os incisivum*, partial loss of the *rugae palatinae* and the dental plate. A sigmoid curvature of the rostral lower jaw as well as a lateral deviation of the face and rostral lower jaw was highly variable in their expression. These deformations were present in eight of nine cases. In addition to the complete CLJ, three animals had an incomplete CLJ on the contralateral site with variable defects of the rostral part of the *proc. nasalis* of the *os incisivum.*

**Conclusions:**

A complete CLJ is obviously accompanied by a loss of parts of the *proc. nasalis* of the *os incisivum*. Extent and localization of the missing parts of the *proc. nasalis* were similar in all cases. A precise classification of the various CLJ forms is necessary.

**Electronic supplementary material:**

The online version of this article (doi:10.1186/s13028-015-0177-0) contains supplementary material, which is available to authorized users.

## Background

Non-syndromic congenital cleft lip and jaw (CLJ) is rarely reported in cattle. Cases of CLJ have been reported in Austrian Simmental, Charolais, Czech Red Pied, German Holstein, Gir, Hereford, Japanese Brown, Shorthorn and Vorderwald × Montbéliarde cattle [1–14]. According to the location and morphology, CLJ can be classified into three main groups, i.e. unilateral CLJ on the right or left side, median CLJ and bilateral CLJ with a cleft palate. In addition, syndromic forms show bilateral CLJ with malformations in other organs [11, 13].

CLJ is generally diagnosed easily by the externally obvious lesions in the lip and upper jaw, but presence and grade of lesions vary among cases when examined in detail. Koch et al. [15] described orofacial malformations in humans and developed a system to record morphological details thus allowing accurate phenotyping. Accurate phenotyping is essential for understanding the epidemiology and etiology of congenital malformations, particularly, if phenotypic expression varies [16] and precise recording is necessary to be able to distinguish among closely related phenotypes [17]. In human medicine, different systems with descriptors and symbolic representations for orofacial malformations exist [15, 18–24].

Three cases of unilateral CLJ have been ascertained in Vorderwald × Montbéliarde cattle in 2009–2010 [13] and further six cases were reported by farmers in the following years. CLJ was not known in this breed before and the frequency of this condition was seemingly increasing. The objective of the present study was to develop a classification system for CLJ in Vorderwald × Montbéliarde cattle for fast characterization of the anomaly as well as ensuring a consistent recording of the underlying defects.

## Methods

Nine Vorderwald × Montbéliarde cattle (cases 1–9) showing congenital CLJ had been sampled from 2009 to 2013, including three cases reported by Lupp et al. [13].

The sex ratio was 4 males and 5 females (0.8:1), the calves were born on different farms and were the first cases of CLJ in the respective herds according to the owners. All cases with exception of case 2 underwent a detailed examination based on either computed tomography (CT) scanning (cases 3–9) or digital radiography (case 1). Age at CT scanning was 3–6 months and 12 months at radiology. The CT scanning image acquisition was performed under general anaesthesia with the animal positioned in sternal recumbency on the CT table. CT scans were acquired with a multislice helical CT scanner (Brilliance-™-CT BigBore, Oncology, Philips Healthcare, Best, The Netherlands) using conventional settings (90 kV, 100 mAs) and 2 mm slice thickness.

The study was conducted according to national and international guidelines for animal welfare. The study was approved by the Institutional Animal Care and Use Committee of Lower Saxony, the State Veterinary Office Niedersächsisches Landesamt für Verbraucherschutz und Lebensmittelsicherheit, Oldenburg, Germany (Registration Number 33.9-42502-04-12/1036).

The classification system of CLJ was based on examination of five orofacial structures: (1) lips, (2) the *processus* (*proc.*) *nasalis* of the *os incisivum*, (3) the dental plate with adjacent segments of the hard palate, (4) the facial bones (*os incisivum*, *os maxillare*, *os nasale* and *os palatinum*) and (5) the mandibles. Each structure was given a score reflecting the degree of the lesion from absent (score 0) to severe. We distinguished the site of the cleft lip (SITE), the depth of cleft lip (DEPTH), the width of cleft lip (WIDTH), changes at the dental plate and hard palate (DEN-HAP), the deviation of the face from the midline (DEVIATION), the lateral curvature of the mandibles (CURVATURE), the slope of the anterior part of the lower jaw (SLOPE) and the extend of loss of the *proc. nasalis* of the *os incisivum* (INCISIVUM). A detailed description of the scoring system is provided in Additional file 1.

## Results

**Fig. 1:**
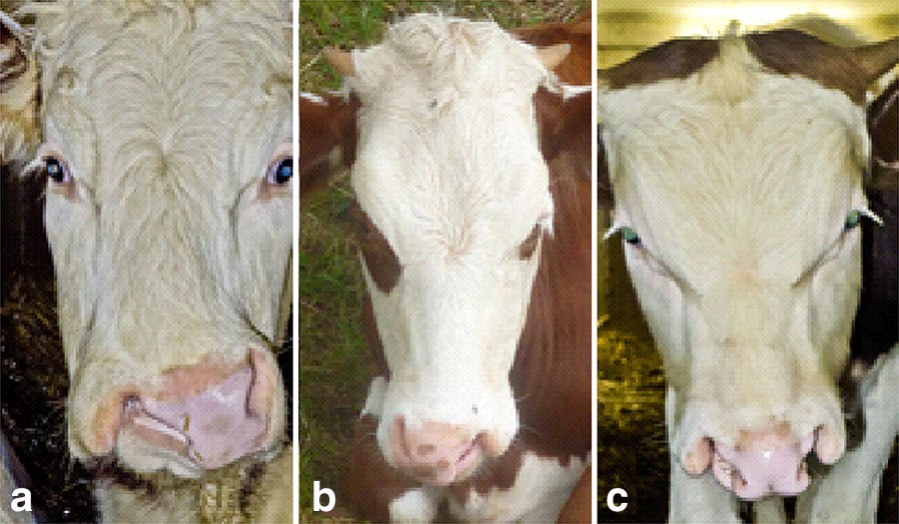
Vorderwald × Montbéliarde cattle showing complete cleft lip and jaw (CLJ). **a** Case 3 shows a complete *right*-sided CLJ with a lateral curvature of the mandibles to the *left* side. **b** Case 8 shows a complete *left*-sided CLJ without lateral curvature of the mandibles. **c** Case 6 shows a complete bilateral CLJ with a slight lateral curvature of the mandibles to the *left* side

**Fig. 2:**
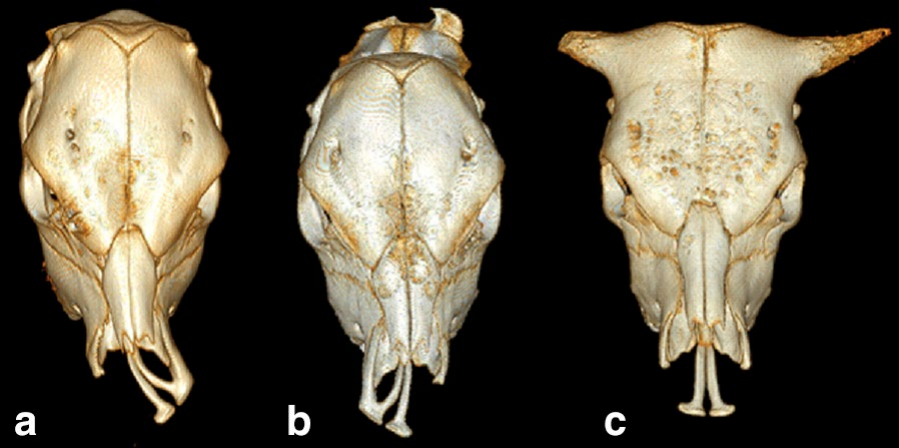
Computed tomography scanning images of the head for Vorderwald × Montbéliarde cattle. **a** Case 3 with a complete *right*-sided cleft lip and jaw, **b** case 8 with a complete *left*-sided CLJ and **c** case 6 with a complete bilateral CLJ. Dorsal view of the skull excluding the mandibles

**Fig. 3:**
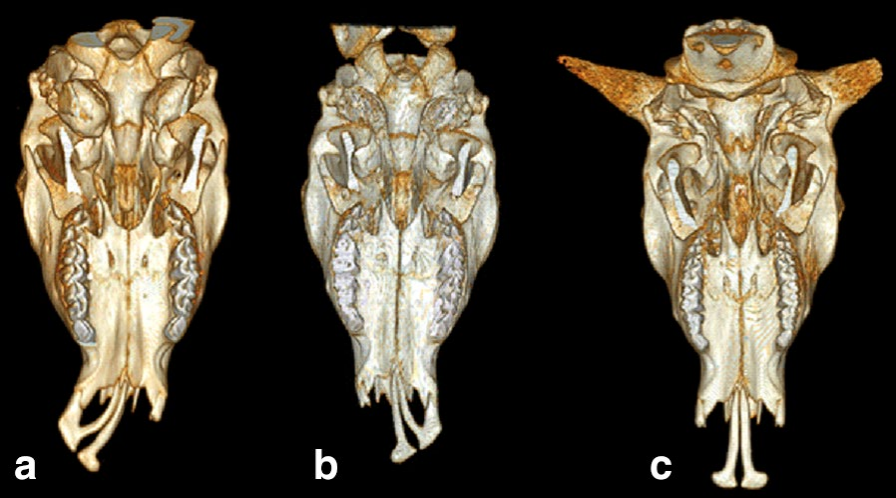
Computed tomography scanning images of the head for Vorderwald × Montbéliarde cattle showing the ventral view of the skull excluding the mandibles. **a** Case 3 shows a complete *right*-sided CLJ. **b** Case 8 shows a complete *left*-sided CLJ. **c** Case 6 shows a complete bilateral CLJ

**Fig. 4:**
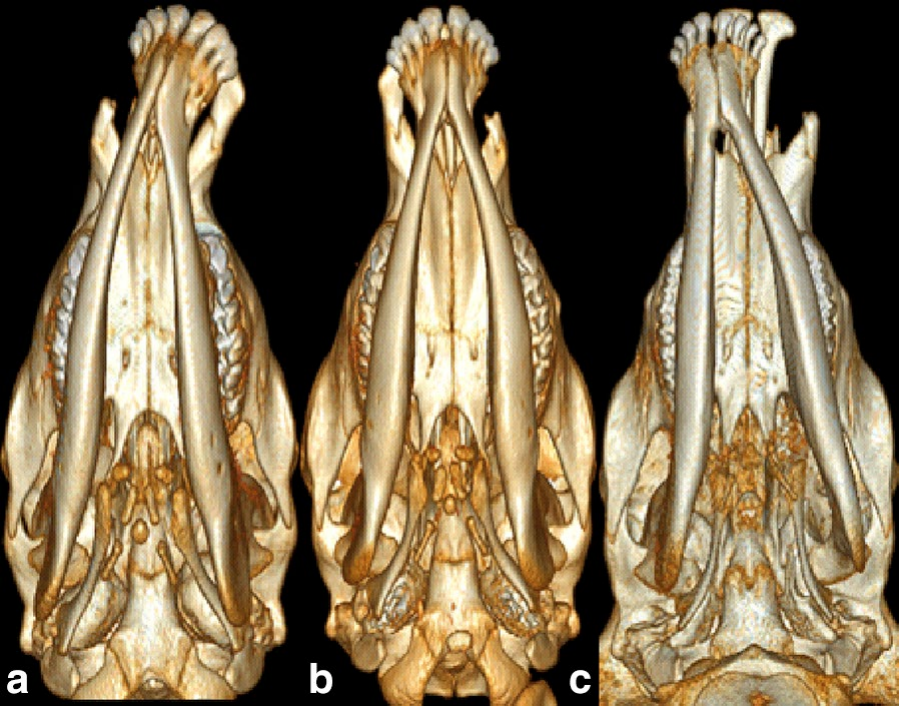
Computed tomography scanning images of the head for Vorderwald × Montbéliarde cattle affected by cleft lip and jaw (CLJ) showing the lateral curvature of the mandibles. **a** Case 3 shows a complete *right*-sided CLJ with a lateral curvature of the mandibles to the *left* side. **b** Case 8 shows a complete *left*-sided CLJ without lateral curvature of the mandibles. **c** Case 6 shows a complete bilateral CLJ with a slight lateral curvature of the mandibles to the *left* side

All cases had complete unilateral or bilateral CLJ (Table 1), while median CLJ was not found. In two cases with a left-sided complete CLJ, and in one case with a right-sided complete CLJ, an incomplete cleft lip on the contralateral side was seen (Figs. 1, 2, 3, 4). An overview of the classification of the cases is given in Table 2.

**Table 1:** Description of the cleft lip and jaw (CLJ) cases in nine Vorderwald × Montbéliarde cattle

Case no.	Orofacial cleft				Sex	
	Complete CLJ		Incomplete CLJ		Male	Female
	Right	Left	Right	Left		
1	+				+	
2	+					+
3	+					+
4		+	+		+	
5	+					+
6	+	+			+	
7	+			+		+
8		+				+
9		+	+		+

**Table 2:** Scores according to the classification system for cleft lip and jaw in nine Vorderwald × Montbéliarde cattle

Case	SITE	DEPTH	WIDTH	DEN-HAP	DEVIATION		CURVATURE		SLOPE		INCISIVUM
					To left	To right	To left	To right	Left-to-right	Right-to-left	
Score range	0–4	0–5	0–6	0–9	0–3	0–3	0–3	0–3	0–3	0–3	0–9
1	1	5	3	2	3			x	3		5
2	1	5	3	2	3			x	3		5
3	1	5	4	2	3			3	2		5
5	1	5	3	2	3			2	3		5
7	1	5	5	2	2			1		0	5
		3	4								1
8	2	5	2	2		1		0		1	5
4	2	5	2	2		2	3			1	5
		3	4								3
9	2	5	4	2		2	2			2	5
		2	6								2
6	4	5	1	2	1		3		2		5
		5	1								5

The nine CLJ-affected Vorderwald × Montbéliarde cattle were scored for the site of cleft lip (SITE), depth of cleft lip (DEPTH), width of cleft lip (WIDTH), changes of the dental plate and hard palate (DEN-HAP), deviation of the face from the midline (DEVIATION), degree of the lateral curvature of the mandibles (CURVATURE), slope of the anterior part of the lower jaw (SLOPE) and bone loss at the *proc. nasalis* of the *os incisivum* (INCISIVUM). Score 0 represents no lesions, while increasing scores reflect increasing severity. See Additional file 1 for details on the scoring system. A right-sided minor form and a right-sided major-form of a cleft lip with a vertical dimension <1/2 accompanied cases 4 and 9 with a complete left CLJ. Case 7 with a complete right-sided CLJ had an incomplete cleft lip with a vertical dimension <1/2 on the left side. Animals with a complete right-sided CLJ (cases 1, 2, 3, 5) exhibited the highest degree of deviation of the face including the mandibles. Cases with a complete unilateral CLJ and a minor or major form of cleft lip on either side (cases 4, 7, 9) had a moderate lateral deviation of the face and mandibles. Complete left-sided (case 8) and complete bilateral CLJ (case 6) were associated with the mildest degree of deviation of the face

**Fig. 5:**
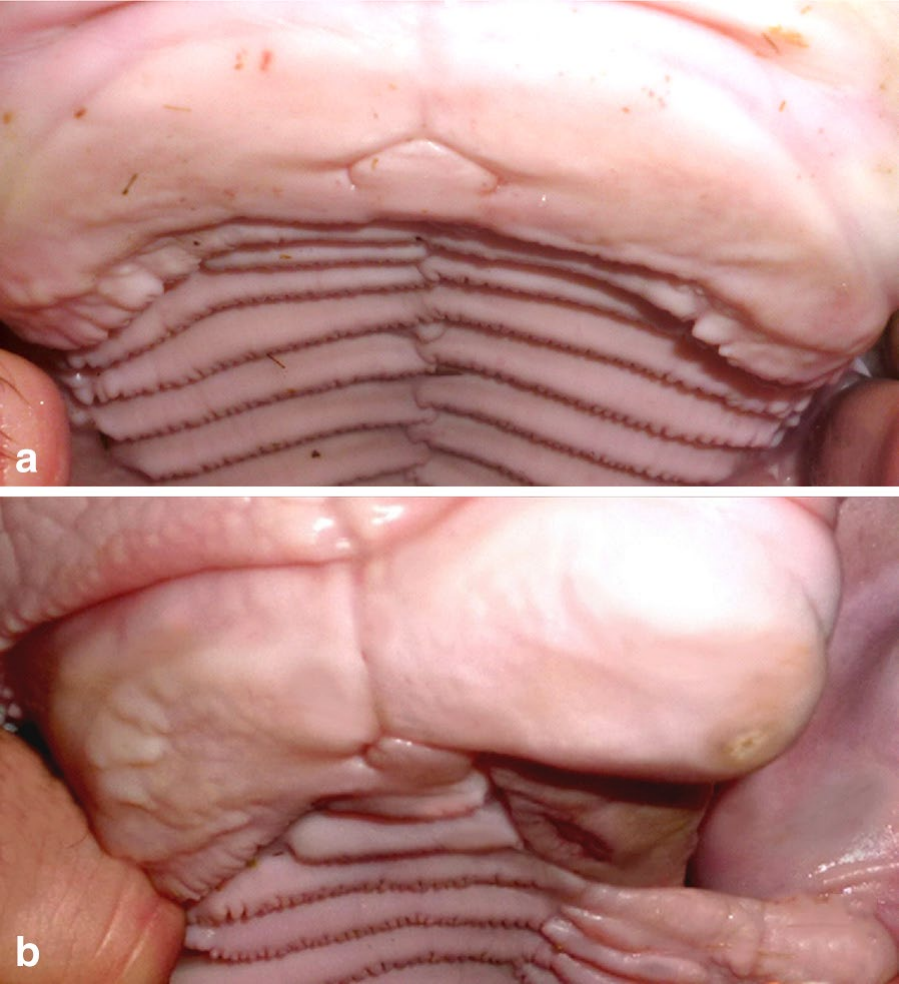
Roofs of the oral cavity of a control and a cleft lip and jaw (CLJ) affected Vorderwald × Montbéliarde cattle. **a** Normally formed roof of an oral cavity of a control without a CLJ. In the foreground, the *papilla incisiva* is visible on the dental plate. Behind the dental plate, the connection to the hard palate and the *rugae palatinae* on both sides are visible. **b** The oral cavity of Vorderwald × Montbéliarde cattle (case 8) with a *left*-sided CLJ is shown. Approximately 1/3 of the dental plate is missing on the *left*. On the adjacent hard palate, *rugae palatinae* are missing

Absence of about 1/3 of the dental plate on the ipsilateral side of the CLJ was observed in all cases. Minor and major forms of cleft lips were not accompanied by a partial loss of the dental plate. On the hard palate adjacent to the complete CLJ, missing *rugae palatinae* were noticed in all cases (Fig. 5). The lateral deviation of the face and mandibles concomitantly occurred consistently but in varying degrees in all cases.

A sigmoid flexure of the rostral part of the mandibles was identified in all cases except case 8 (Fig. 4). This curvature had the steepest slope in case 6 with a complete bilateral CLJ and in the right-sided complete CLJ cases 1, 2, 3 and 5. The largest angular deviation of the occlusal plane was also found in the right-sided CLJ cases and the bilateral CLJ case.

In all cases, the same segment of the *proc. nasalis* was missing on the ipsilateral side as the complete CLJ. This segment includes the rostral abaxial part of the *os incisivum*. Further changes of the *proc. nasalis* in the presence of incomplete cleft lips were located in the middle of the rostral abaxial part of the *proc. nasalis*. In case 9, a small notch ventrally at the *proc. nasalis* corresponded to the minor form of a cleft lip (Additional file 2). A large notch of 2.5 × 0.8 cm in size was present ventrally at the *proc. nasalis* underlying the major form of a cleft lip in case 4 (Additional file 3). Case 7 revealed a ring-like bone loss in the *proc. nasalis* with only a thin bone bridge remaining (Additional file 4).

## Discussion

In Vorderwald × Montbéliarde cattle phenotypic expression of CLJ includes unilateral (right- or left-sided) and bilateral cases. In cases with a unilateral complete CLJ, the abaxial rostral part of the *proc. nasalis*, approximately 1/3 of the dental plate and the *rugae palatinae* of the adjacent hard palate were missing. All cases examined using X-rays or CT scanning (n = 8) had similar changes of the *os incisivum*, the dental plate and the hard palate in the ipsilateral to the complete CLJ. The deficit of the middle segment of the *proc. nasalis* was consistently observed in these cases ipsilateral to the complete CLJ. Rieke [6] also described the absence of this segment of the *proc. nasalis* in German Holstein calves but also a progression of this defect through the middle of the corpus of the premaxilla. The macerated heads of CLJ cases reported by Moritomo et al. [11] also showed a partial loss of the *proc. nasalis*. A female Japanese Brown calf with a left-sided CLJ missed this segment. Furthermore, the outward curvature of the *os incisivum* was also associated with unilateral CLJ on the contralateral side of the muzzle in the cases presented by Moritomo et al. [11].

In agreement with Rieke [25], unilateral CLJ was most frequent. Unilateral CLJ is also most common in humans and laboratory rodents [26, 27]. In six CLJ affected Japanese Brown cattle, three had unilateral CLJ, one had bilateral CLJ and two had median CLJ [11]. Right-sided CLJ were more frequent than left-sided in the present study. Rieke [25] observed 33.3 % right-sided CLJ and 21.7 % left-sided CLJ which is in contrast to humans and laboratory animals having a majority of left sided CLJ [26, 27].

The term “cleft lip” includes complete and incomplete clefts of the upper lip independent of whether the maxillary alveolus is affected [28]. In human medicine, notches <3 mm were defined as microforms [29]. In microform clefts the functional tissue layer such as the muscle for the lip, cartilage of the nose and the septum or bone of the maxillary alveolus are not affected [15]. Minor forms in humans include defects extending 3 mm or more but <1/2 of the vertical dimension of the lip and the nostril floor [29]. A complete cleft is defined as a cleft which includes the upper lip and nostril floor in total [30], while an incomplete cleft is defined as involving only a part of the upper lip. Incomplete cleft lips on the contralateral side were present in 3/9 of the affected Vorderwald × Montbéliarde cattle. This could have particular importance for ascertainment of possible cases with incomplete cleft lip. Mayr et al. [5] described a three-years-old Fleckvieh bull with an incomplete cleft lip. This Austrian bull sired 55 descendants of which 32 had a complete CLJ. Therefore, in cattle each cleft lips should be registered as a mild form of a CLJ. Phenotypic variants like lip pits, prints or microforms of cleft lips should be considered as an anomaly and may have consequences for selective breeding, particularly, if relatives with CLJ are observed.

On the side with the complete CLJ, the size of the missing parts of the dental plate and of the hard palate was very similar in all cases. Swartz et al. [7] studied two Angus calves with unilateral left-sided CLJ with variable defects of the dental plate and hard palate. The dental plate was completely missing on the left side and a segment on the right side lacked in one case. Furthermore, a V-shaped wedge of the hard palate lacked in this case. The second case was lacking approximately the same proportion of the dental plate as observed in Vorderwald × Montbéliarde cattle.

The curvature of the lower jaw may be a secondary deformation that develops due to the absence of the pressing surface between the mandibles and the missing segment of the *os incisivum* contralateral to the CLJ [10]. However, among the nine cases, the bull with a complete bilateral CLJ (case 6) showed the most pronounced rostral curvature of the mandibles. This latter case is apparently in contrast to the assumption of an induced malformation through the missing pressure of the upper jaw on this part of the mandibles.

Lateral deviation of the face was obviously associated with CLJ as reported previously [7, 10, 25] and in all the present cases. A severe deviation of the face may cause respiratory distress due to reduced size of the nostril and air-conducting ways. Thielscher [10] reported unilateral nasal obstruction and dyspnea in a Red and White Spotted calf with right-sided CLJ. One case with a left sided CLJ mentioned by Swartz et al. [7] showed extreme right curvature of the rostral end of the nose and muzzle. In this case, the dorsal alar cartilage on the left nostril dropped ventrally and reduced further the opening to the nares. The consequences of the lateral deviation of the face in the affected cattle were limited to dental anomalies in form of wiggling teeth with sporadic tooth loss, probably due to pathological friction. This was particularly noticed in the bull with a complete left-sided CLJ and a right-sided minor form (case 9) at an age of one year and in the two right-sided CLJ cows (cases 3 and 5) when they reached an age of 3 and 4 years, respectively.

## Conclusions

Complete CLJ was accompanied by loss of parts of the *proc. nasalis* of the *os incisivum.* The extent and localization of the missing parts of the *proc. nasalis* were similar in all cases. The accompanying orofacial defects like lateral deviation of the face and rostral lower jaw as well as the sigmoid curvature of the rostral lower jaw showed a high degree of variability. A precise documentation of the changes, e.g. based on CT scanning, facilitates precise identification of different forms of CLJ and correct classification of animals with morphologically identical orofacial malformations for further analyses.

## Additional files

10.1186/s13028-015-0177-0 Explanation of the scoring system for cleft lips and accompanying malformations in the face. The system distinguishes the site of the cleft lip (SITE), the depth of cleft lip (DEPTH), the width of cleft lip (WIDTH), changes at the dental plate and hard palate (DEN-HAP), the deviation of the face from the midline (DEVIATION), the lateral curvature of the mandibles (CURVATURE), the slope of the anterior part of the lower jaw (SLOPE) and the extend of loss of the *proc. nasalis* of the *os incisivum* (INCISIVUM).
10.1186/s13028-015-0177-0 a) The picture shows the muzzle of case 9 with a complete left-sided cleft lip and a minor form on the right side of the lip. b) Computed tomography scanning image of the head showing the lateral view of the skull. The arrow marks the deficit of bony substance (25 %) ventral to the bone shaft corresponded to the minor form of a cleft lip.
10.1186/s13028-015-0177-0 a) The picture shows the muzzle of case 4 with a complete left-sided cleft lip and a macroform on the right side of the lip. b) Computed tomography scanning image of the head showing the lateral view of the skull. The arrow marks the deficit of bony substance (50 %) ventral to the bone shaft corresponded to the macroform of a cleft lip.
10.1186/s13028-015-0177-0 a) The picture shows the muzzle of case 7 with a complete right-sided cleft lip and a macroform on the left side of the lip. b) Computed tomography scanning image of the head showing the lateral view of the skull. The arrow marks the deficit of bony substance in form of a ring-like bone loss corresponded to the macroform of a cleft lip.

## Abbreviations

CLJ: cleft lip and jaw
CT: computer tomography
CURVATURE: lateral curvature of the mandibles
DEN-HAP: dental plate and hard palate
DEPTH: depth of cleft lip
INCISIVUM: *proc. nasalis* of the os incisivum
Proc: *processus*
DEVIATION: deviation of the face from the midline
SLOPE: slope of the anterior part of the lower jaw
WIDTH: width of cleft lip
