# Supplementary material for: Phenotypic classification of variability of non-syndromic congenital cleft lip and jaw in Vorderwald × Montbéliarde cattle
Source: Acta Vet Scand. 2015 Dec 15;57:87. doi: 10.1186/s13028-015-0177-0 (PMC4678477; doi:10.1186/s13028-015-0177-0)
Supplement: Supplementary file 1 — 10.1186/s13028-015-0177-0 Explanation of the scoring system for cleft lips and accompanying malformations in the face. The system distinguishes the site of the cleft lip (SITE), the depth of cleft lip (DEPTH), the width of cleft lip (WIDTH), changes at the dental plate and hard palate (DEN-HAP), the deviation of the face from the midline (DEVIATION), the lateral curvature of the mandibles (CURVATURE), the slope of the anterior part of the lower jaw (SLOPE) and the extend of loss of the proc. nasalis of the os incisivum (INCISIVUM). [file 13028_2015_177_MOESM1_ESM.pdf]

Additional file 1

Explanation of the scoring system for cleft lips and accompanying malformations in the face. The system distinguishes the site of the cleft lip (SITE), the depth of cleft lip (DEPTH), the width of cleft lip (WIDTH), changes at the dental plate and hard palate (DEN-HAP), the deviation of the face from the midline (DEVIATION), the lateral curvature of the mandibles (CURVATURE), the slope of the anterior part of the lower jaw (SLOPE) and the extent of the loss of the *processus* (*proc.*) *nasalis* of the o*s incisivum* (INCISIVUM).

Site of the cleft lip (SITE)

Scoring the site of cleft lip	Description
1	Unilateral right
2	Unilateral left
3	Median
4	Bilateral left and right

These changes are visible in animals affected by CLJ. These changes can occur unilaterally on the right or left site of the muzzle, bilaterally or in the median plane.

Depth of cleft lip (DEPTH)

Scoring the depth of cleft lip	Designation	Description
0		No change
1	Cleft lip <1/4 of its dimension	Micro form
2	Cleft lip =1/4	Minor form
3	Cleft lip =2/4	Moderate
4	Cleft lip =3/4	Severe
5	Cleft lip =4/4	Complete

The term cleft lip includes complete and incomplete clefts of the upper lip independent of whether the maxillary alveolus is affected. The manifestation of this malformation varies in a broad spectrum. For cattle, we propose to distinguish five classifications with subdivisions in the range of 1/4 of the dimension of the upper lip. Microforms are the mildest form and include clefts <1/4 of the dimension of the upper lip and the nostril floor. The minor form of clefts accounts for about 1/4 of the vertical dimension of the lip and the nostril floor in cattle.

If the cleft extends to 2/4 or more of the vertical dimension obvious separations of tissue can be observed. Thus, cleft lips >1/4 of the vertical dimension of the muzzle can be subsumed under major forms.

Width of cleft lip (WIDTH)

Scoring the width of cleft lips	Description
0	No change
1	0-20°
2	21-40°
3	41-60°
4	61-80°
5	81-100°
6	>100°

Width of clefts should be measured to determine whether clefts with identical length differ in width and therefore should be evaluated as more severe forms. The angle formed by the cleft should be recorded at the *sulcus alaris*.

Changes at the dental plate and hard palate (DEN-HAP)

Scoring the changes at the dental plate and hard palate	Description
0	No change
1	About 1/3 of the dental plate is missing
2	About 1/3 of the dental plate is missing and adjacent *rugae palatinae* of the hard palate are changed or not present
3	About 1/3 of the dental plate and adjacent segments of the hard palate are missing
4	About 2/3 of the dental plate is missing
5	About 2/3 of the dental plate is missing and adjacent *rugae palatinae* of the hard palate are changed or not present
6	About 2/3 of the dental plate and adjacent segments of the hard palate are missing
7	About 3/3 of the dental plate is missing

8	About 3/3 of the dental plate is missing and adjacent *rugae palatinae* of the hard palate are changed or not present
9	About 3/3 of the dental plate and adjacent segments of the hard palate are missing

Segmental absence of the dental plate leads together with the absence of a part of the *proc. nasalis* to an incomplete separation of the oral and nostril cavity. Therefore, the nasal septum is visible at raised lips. The size of the missing segment of the dental plate and changes in the adjacent hard palate should be taken into account by inspection of the oral cavity roof.

Changes at the os incisivum, os maxillare, os nasale and os palatinum including deviations of the face from the midline (DEVIATION), lateral curvature of the mandibles (CURVATURE) and slope of the anterior part of the lower jaw (SLOPE)

Scoring deviation of the face, lateral curvature of the mandibles and slope of the anterior part of the lower jaw			
Score	DEVIATION	CURVATURE	SLOPE
0	No change	No change	No change
1	Mild 1-5°	Mild	Mild 1-10°
2	Moderate 6-10°	Moderate	Moderate 11-20°
3	Severe >10°	Severe	Severe >21°

The deviation from the midline of the face (DEVIATION) describes the asymmetry of the head to the opposite side of the cleft which concerns mainly the front area of the skull. Deviation of the face is a striking feature that is particularly noticed in combination with unilateral CLJ. These deformations include the *os incisivum*, *os maxillare*, *os nasale*, and *os palatinum*. The lateral deviation of the face is classified into mild, moderate and severe.

The degree of lateral curvature of the anterior part of the mandibles (CURVATURE) is classified into mild, moderate and severe. The degree of the deviation from the median axis can be evaluated by inspection of the anterior part of the mandibles, computed tomography of the mandibles as well as macerated heads.

The sigmoid flexure of the lower jaw (SLOPE) is assessed comparing the level of the teeth on the right and left side of the rostral part of the lower jaw. An abnormal angulation of the occlusal plane is more evident the greater the skeletal malocclusions are. A differentiation among the rise of the lower jaw from the right to the left (right-to-left) or vice versa (left-to-

right) can be made.

Changes only visible using X-ray or computed tomography at the proc. nasalis of the os incisivum (INCISIVUM)

Scoring the loss of the *proc. nasalis* of the *os incisivum*	Description
0	No change
1	Ring-like bone loss
2	25% loss of bony substance ventrally at the *proc. nasalis*
3	50% loss of bony substance ventrally at the *proc. nasalis*
4	75% loss of bony substance ventrally at the *proc. nasalis*
5	Absence of the apical segment of the *proc. nasalis*
6	Absence of the middle segment of the *proc. nasalis*
7	Absence of the caudal segment of the *proc. nasalis*
8	Absence of the apical and middle segment of the *proc. nasalis*
9	Absence of the apical, middle and caudal segment of the *proc. nasalis*

Herein, we distinguish different degrees of the loss of the *proc. nasalis* of the *os incisivum*. The scores 1-4 refer to cases with an incomplete loss of the bony substance of the *proc. nasalis* of the *os incisivum*, scores 5-8 to cases with a complete loss of the bony substance at different degrees and score 9 to cases with a complete absence of the *proc. nasalis* of the *os incisivum*.
