# Supplementary material for: Phenotypic classification of variability of non-syndromic congenital cleft lip and jaw in Vorderwald × Montbéliarde cattle
Source: Acta Vet Scand. 2015 Dec 15;57:87. doi: 10.1186/s13028-015-0177-0 (PMC4678477; doi:10.1186/s13028-015-0177-0)

Additional file 3

a) The picture shows the muzzle of case 4 with a complete left-sided cleft lip and a macroform on the right side of the lip. b) Computed tomography scanning image of the head showing the lateral view of the skull. The arrow marks the deficit of bony substance (50%) ventral to the bone shaft corresponded to the macroform of a cleft lip.

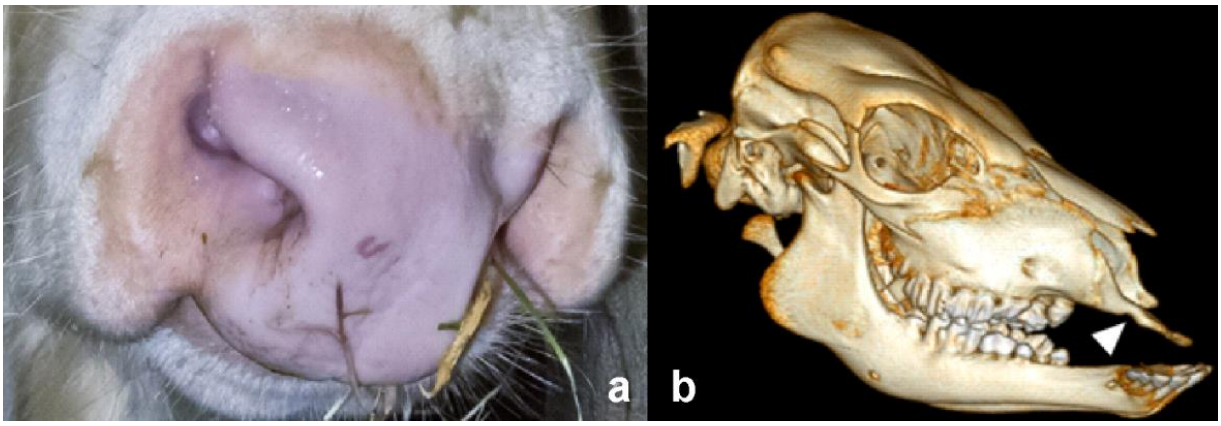

Supplement: Supplementary file 3 — 10.1186/s13028-015-0177-0 a) The picture shows the muzzle of case 4 with a complete left-sided cleft lip and a macroform on the right side of the lip. b) Computed tomography scanning image of the head showing the lateral view of the skull. The arrow marks the deficit of bony substance (50 %) ventral to the bone shaft corresponded to the macroform of a cleft lip. [file 13028_2015_177_MOESM3_ESM.pdf]
