# Supplementary material for: Phenotypic classification of variability of non-syndromic congenital cleft lip and jaw in Vorderwald × Montbéliarde cattle
Source: Acta Vet Scand. 2015 Dec 15;57:87. doi: 10.1186/s13028-015-0177-0 (PMC4678477; doi:10.1186/s13028-015-0177-0)

Additional file 4

a) The picture shows the muzzle of case 7 with a complete right-sided cleft lip and a macroform on the left side of the lip. b) Computed tomography scanning image of the head showing the lateral view of the skull. The arrow marks the deficit of bony substance in form of a ring-like bone loss corresponded to the macroform of a cleft lip.

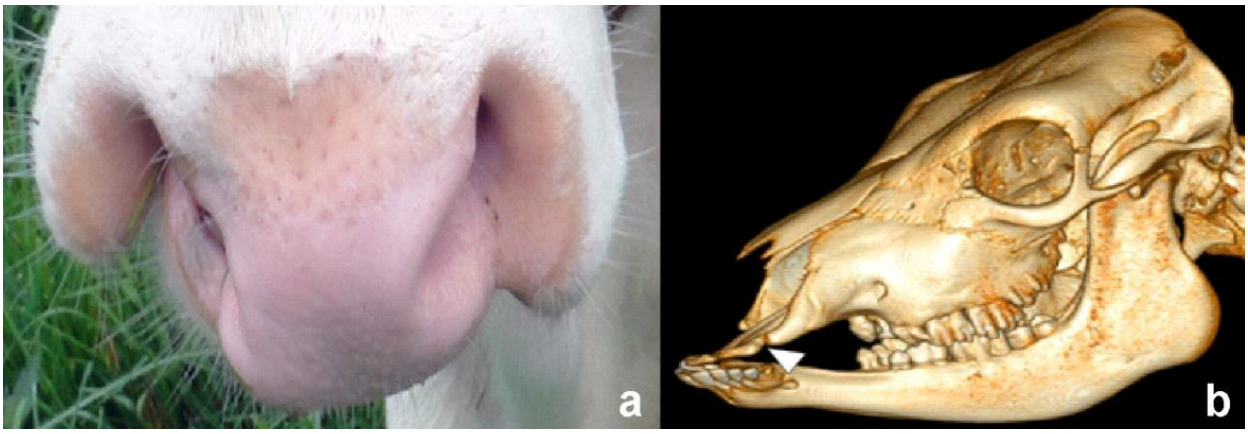

Supplement: Supplementary file 4 — 10.1186/s13028-015-0177-0 a) The picture shows the muzzle of case 7 with a complete right-sided cleft lip and a macroform on the left side of the lip. b) Computed tomography scanning image of the head showing the lateral view of the skull. The arrow marks the deficit of bony substance in form of a ring-like bone loss corresponded to the macroform of a cleft lip. [file 13028_2015_177_MOESM4_ESM.pdf]
